# Synthetic/ECM-inspired hybrid platform for hollow microcarriers with ROS-triggered nanoporation hallmarks

**DOI:** 10.1038/s41598-017-13744-y

**Published:** 2017-10-13

**Authors:** Gesmi Milcovich, Paolo Contessotto, Grazia Marsico, Siti Ismail, Abhay Pandit

**Affiliations:** CÚRAM Centre for Research in Medical Devices, Biomedical Sciences, National University of Ireland, Galway, Ireland

## Abstract

Reactive oxygen species (ROS) are key pathological signals expressed in inflammatory diseases such as cancer, ischemic conditions and atherosclerosis. An ideal drug delivery system should not only be responsive to these signals but also should not elicit an unfavourable host response. This study presents an innovative platform for drug delivery where a natural/synthetic composite system composed of collagen type I and a synthesized polythioether, ensures a dual stimuli-responsive behaviour. Collagen type I is an extracellular matrix constituent protein, responsive to matrix metalloproteinases (MMP) cleavage per se. Polythioethers are stable synthetic polymers characterized by the presence of sulphur, which undergoes a ROS-responsive swelling switch. A polythioether was synthesised, functionalized and tested for cytotoxicity. Optimal conditions to fabricate a composite natural/synthetic hollow sphere construct were optimised by a template-based method. Collagen-polythioether hollow spheres were fabricated, revealing uniform size and ROS-triggered nanoporation features. Cellular metabolic activity of H9C2 cardiomyoblasts remained unaffected upon exposure to the spheres. Our natural/synthetic hollow microspheres exhibit the potential for use as a pathological stimuli-responsive reservoir system for applications in inflammatory diseases.

## Introduction

Reactive oxygen species (ROS) are key pathological signals expressed in inflammatory diseases such as cancer, arteriosclerosis, and reperfusion injury^1^. Oxidative stress in tissues is associated with the overexpression of ROS and represents a relevant hallmark of age-related diseases such as atherosclerosis^2,3^. ROS are intermediate products of the metabolic chain, which leads to the production of ATP, H_2_O and CO_2_. ROS have a disruptive effect not only on the cell membrane by stimulating lipid peroxidation and damaging sulfhydryl bonds, but also at the DNA level^4^. In physiological conditions, tissues can tolerate low levels of hydroxyl radicals. However, during an ischemic-reperfusion event, the anti-oxidant defence system (i.e., superoxide dismutase and catalase enzymes) cannot compensate for the overexpression of these oxidant moieties. Free radical scavengers have been demonstrated to have a preservative effect on rabbit ventricular cardiomyocytes after myocardial infarction^5^. Different sources of ROS have been hypothesized for their release through either the ischemic or the reperfusion phase^6^. However, the use of anti-oxidant molecules in preclinical and clinical trials is still in its infancy, mainly due to the need to elucidate the mechanism of ROS production during the reperfusion phase and the need for a multiple therapeutic strategy to target ROS overexpression^7,8^. For these reasons, the suppression of pro-inflammatory pathways inspires most of current research for addressing inflammatory diseases^9^. Nevertheless, ROS-triggered platforms represent a promising alternative to overcome oxidative damage, while exploiting the same pathological signal for the development of *in-situ* drug delivery technologies^10,11^, which can be tailored for specific targets including dendritic cells^12^ or enriched with peptides to promote a pro-angiogenic host response^13^. A physiological-inspired approach involves the employment of macromolecules which are ROS-responsive but not ROS-degradable, the most promising of these being poly(propylene sulfide) (PPS)^14^. PPS is a highly hydrophobic polymeric backbone, which transitions to a hydrophilic moiety upon oxidation, and it is thus a suitable material for the design of ROS-responsive systems. Specifically, this transition occurs through poly(propylene sulfoxide) and finally poly(propylene sulfone), which promotes an oxidation-triggered swelling switch^15^. The process of ROS self-propagation enables a continuous spatio-temporal release of bioactive therapeutic molecules at the target site. Microspheres^16,17^, hydrogels^18^ and micelles^19^ have been fabricated from triblock and diblock copolymers, comprised of PPS, polyethyetylene glycol (PEG) and polydimetylacrylamide (PDMA). In parallel, different strategies have utilised extracellular matrix proteins specifically collagen type I as reservoir drug delivery platforms exploiting the protein’s responsiveness to matrix metalloproteinase cleavage. Specifically type I collagen, which plays a relevant role in the structural integrity of myocardial tissue is disrupted by the action of matrix metalloproteinases after myocardial infarction (MMP-1)^20,21^. Thus, by combining these different approaches, we hypothesize that PPS-type I collagen hollow microspheres will provide an oxidation-sensitive and matrix-mimicking reservoir system. The objectives of this study were: first, the synthesis of a functionalised polythioether and its incorporation into a type I collagen matrix. Secondly, the design of the optimal conditions to fabricate a composite hollow sphere construct composed of type I collagen/polythioether respectively, to develop a naturally mimicking platform, with specific ROS-responsiveness.

## Results and Discussion

In order to achieve a final hybrid ECM-synthetic polymer construct with ROS-responsive properties, we first synthesised PPS and evaluated its cytotoxicity and its ROS responsiveness over a broad range of concentrations (50 µg/mL–1,000 µg/mL). Mixed collagen/PPS hollow spheres were fabricated and characterized by means of SEM, TEM, AFM, FT-IR and DLS. The formation of pores in the collagen-PPS composite hollow spheres was evaluated after exposure to different physiologically relevant H_2_O_2_ concentrations (100 µM–100 mM) and relevant exposure conditions (up to 72 hours), as described in the materials and methods section. The spheres were characterized for ROS-responsive behaviour via SEM, FT-IR analysis and by characterizing the pore size distribution.

The results presented herein, demonstrate that the hybrid construct possesses the properties required for application under inflammatory conditions, combining the synergistic advantages of its individual components.

### Polypropylene sulfide synthesis, ROS-responsiveness and cytotoxicity

Polypropylene sulfide (PPS) was synthesized by emulsion polymerization, as described in the materials and methods section. The amide adduct was obtained and characterized (Supplementary, Figs S1–S5), and then hydrolyzed to its carboxylic acid in basic conditions. The oxidation of thioether bonds to sulfone under ROS-mimicking conditions (H_2_O_2_) was validated by^1^HNMR and FT-IR (Figs S7–S8) on the amide polypropylene sulfide adduct.

The resulting sulfones exhibited more hydrophilic characteristics because of a strong dipole due to the presence of the sulfur oxygen double bond. The synthesized compound was demonstrated to be highly responsive to ROS as the swelling switch took place at a very low H_2_O_2_ concentration (Table 1), which mimics ROS in the physiological range.

**Table 1:** ROS-mimicking conditions (left) and time points of assessment (right).

H_2_O_2_ concentration	Time points
100 mM	T = 0
1 mM	T = 24 h (day 1)
100 μM	T = 72 h (day 3)

Further cytotoxicity assays were performed, using a linear PEG 2000 as a control. The GPC results, estimated a molecular weight of 3086 for PPS (Fig. S1). While PEG 2000 did not affect cellular metabolic activity, the polypropylene sulfide concentration significantly reduced cellular metabolic activity. Thus, the hollow spheres fabrication protocol was adapted in order to completely eliminate any side product or organic solvent traces: appropriate solvents and washing procedures were employed, as detailed in the materials and methods section. Moreover, the preliminary cytotoxicity assays performed on the PPS polymer allowed us to tune the hollow spheres final concentration range. In addition, further studies carried out in our laboratory demonstrated that the balance between size and concentration is a key factor to modulate the cytotoxic response. Thus, the final hollow spheres concentration was decreased compared to the PPS concentration in the mg/mL range, in order to reduce toxicity constraints.

### Fabrication of hollow spheres

**Figure 1:**
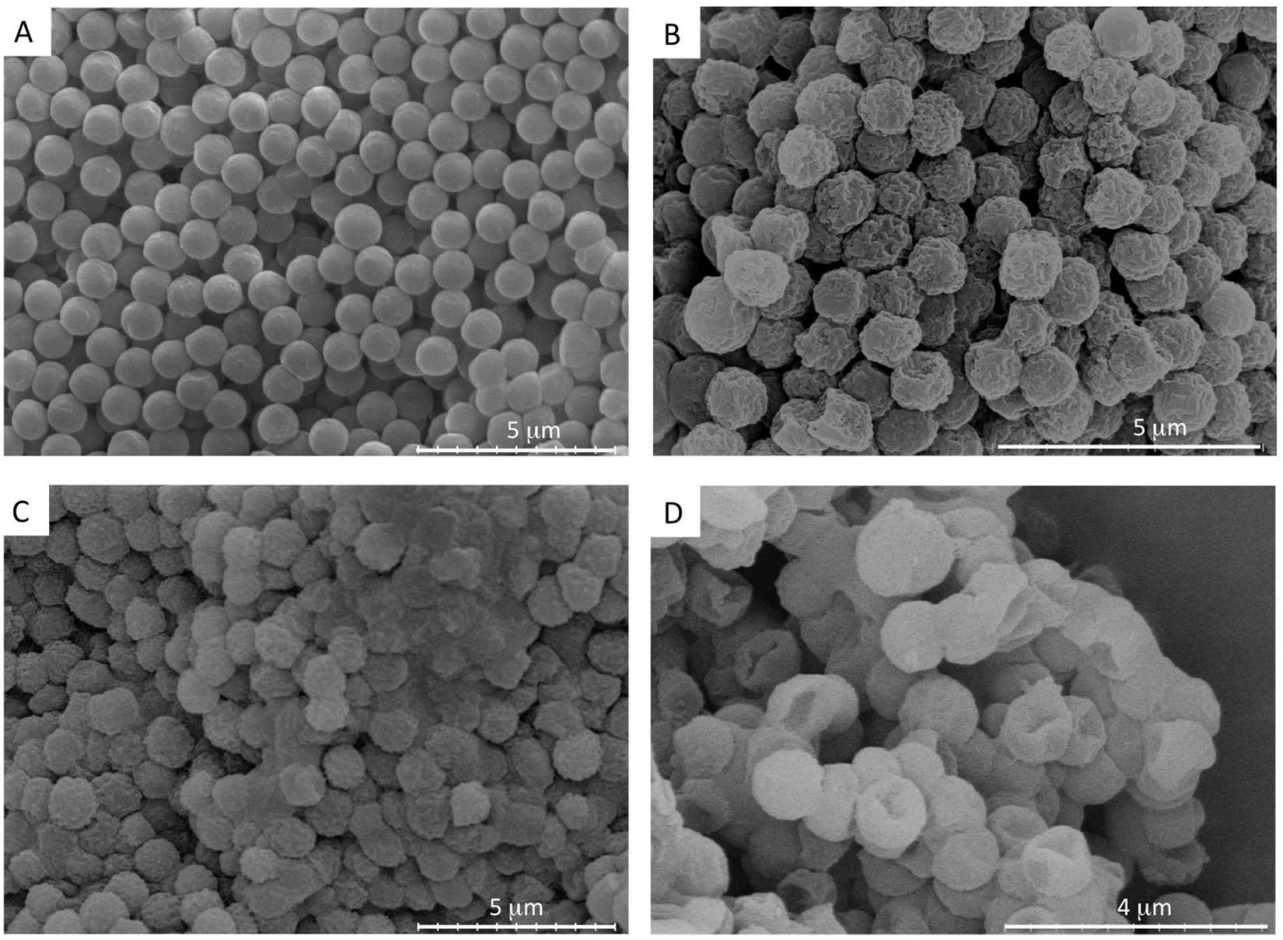
SEM images of hollow spheres. (**A**) 1 μm polystyrene templates, (**B**) mixed collagen-PPS hollow spheres, (**C**) mixed collagen-dithiolPEG hollow spheres (control), (**D**) mixed collagen-4armPEG hollow spheres (control).

**Figure 2:**
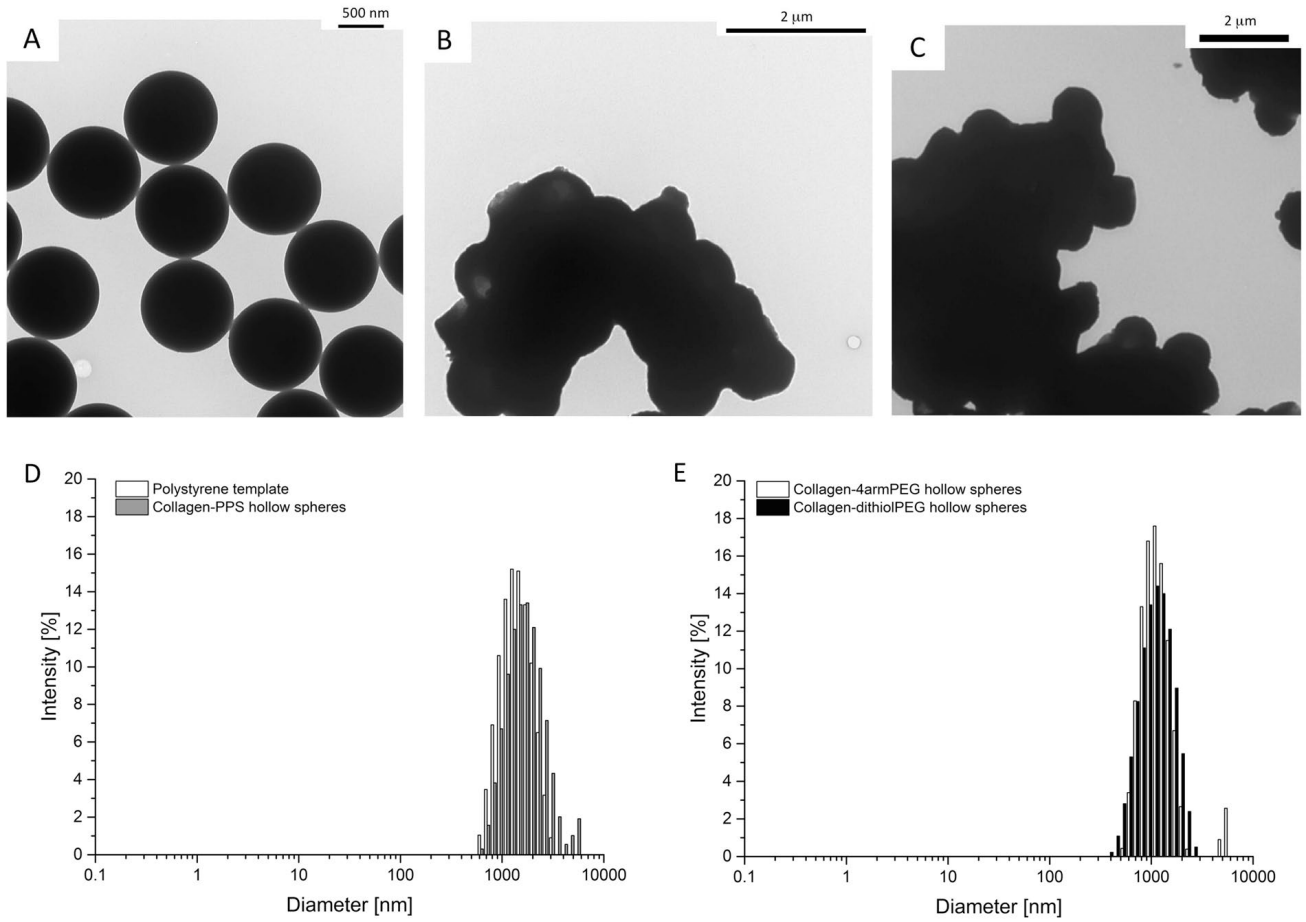
Hollow spheres TEM imaging and DLS analysis. (**A**) 1 μm polystyrene templates, (**B**) mixed collagen-PPS hollow spheres, (**C**) mixed collagen-4armPEG hollow spheres (control). (**D**) DLS plots of size distribution for the 1 μm polystyrene templates and mixed collagen-PPS hollow spheres, E) DLS plots of size distribution for the 1 μm mixed collagen-4armPEG hollow spheres and mixed collagen-dithiolPEG hollow spheres (controls).

The most stable and optimal ratio of concentration of collagen to PPS was found to be 2.5 mg/mL to 100 mg/mL, respectively. Mixed collagen-dithiolPEG and mixed collagen-4armPEG hollow spheres were fabricated, based on this optimized ratio. All tested samples were analyzed for size and were 1 µm in diameter (Figs 1, 2, S9 and S10).

The hollowness of the spheres and their shape, size and distribution characteristics were validated by TEM and DLS analysis (Fig. 2). The spherical hollow spheres exhibited a uniform diameter of 1 μm. The two step coating over the polystyrene template guaranteed that a collagen (first layer, natural polymer) was cross-linked by the PPS (or 4armPEG or dithiolPEG, synthetic component). The polystyrene template was removed by THF. Both microscopy and DLS data demonstrated that the obtained hollow spheres self-assembled into macro-aggregates (Fig. 2). The same phenomenon was observed for the polystyrene sacrificial template, which was easily resolved by gentle tip-sonication. SEM and TEM analysis revealed a higher self-assembly into macro-aggregates for the hollow spheres. Homogeneously distributed hollow spheres were reconstituted by carefully sonicating the solution sample, as confirmed by DLS analysis. Moreover, the hollow spheres showed an interesting rough surface, which was assessed by SEM, TEM and further AFM analysis (Figs 1, 2 and S6).

**Figure 3:**
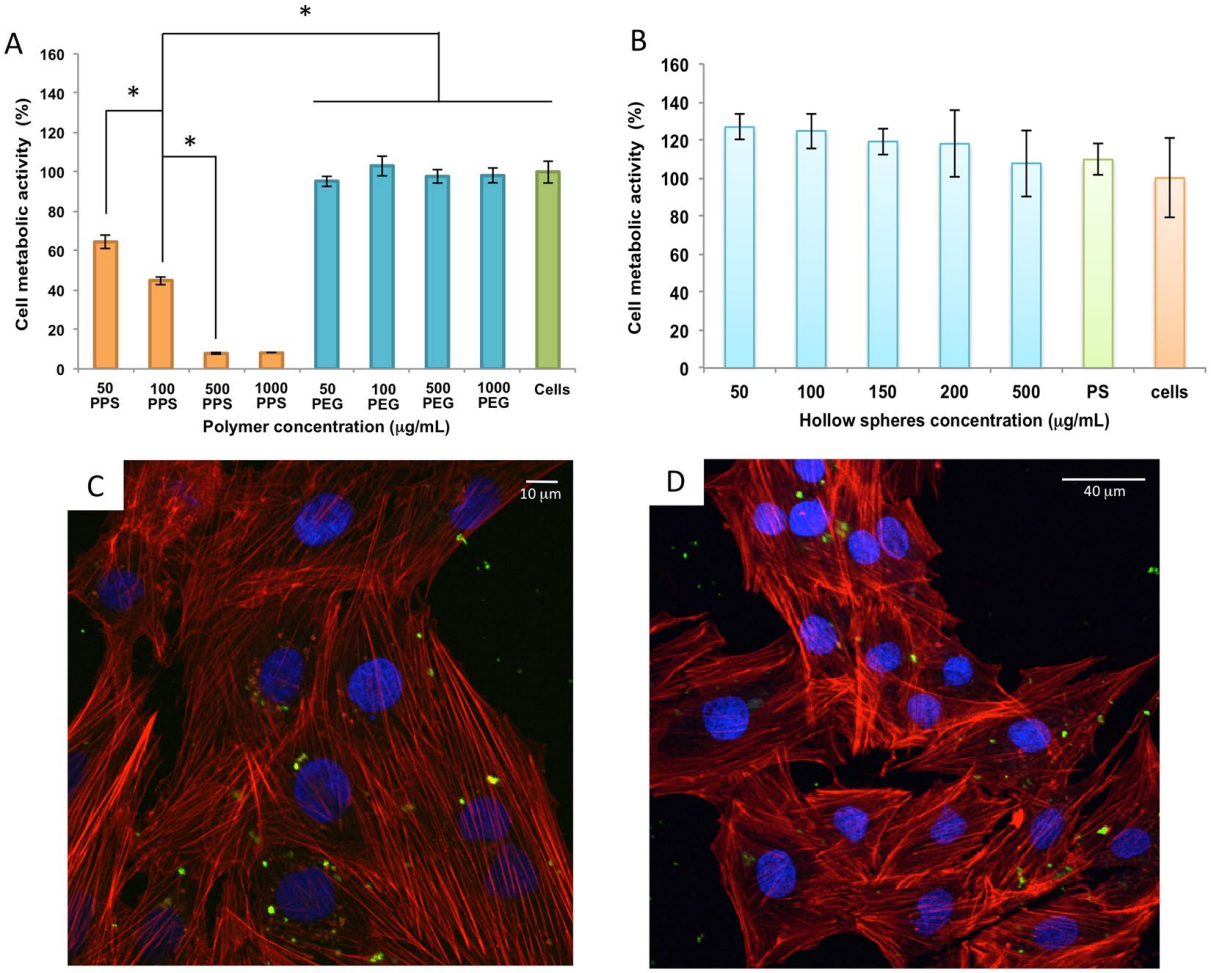
Cell metabolic activity on rat cardiomyoblasts H9C2 cells, after 24 hours and confocal imaging after microspheres internalization. alamarBlue® assay was carried out on: (**A**) the synthesized polypropylene sulfide (PPS) in orange, PEG2000 (blue), untreated cells (green); (**B**) collagen-PPS hollow spheres (light blue), polystyrene (green) and untreated cells (orange) were used as controls (n=3, p < 0.05). Confocal images of H9C2 rat cardiomyoblasts incubated for 24 hours with FITC-labeled collagen-PPS hollow spheres (green) at 50 μg/mL (**C**); and 100 μg/mL (**D**). Nuclei were stained with DAPI (blue), while actin fibers were stained with rhodamine phalloidin (red).

**Figure 4:**
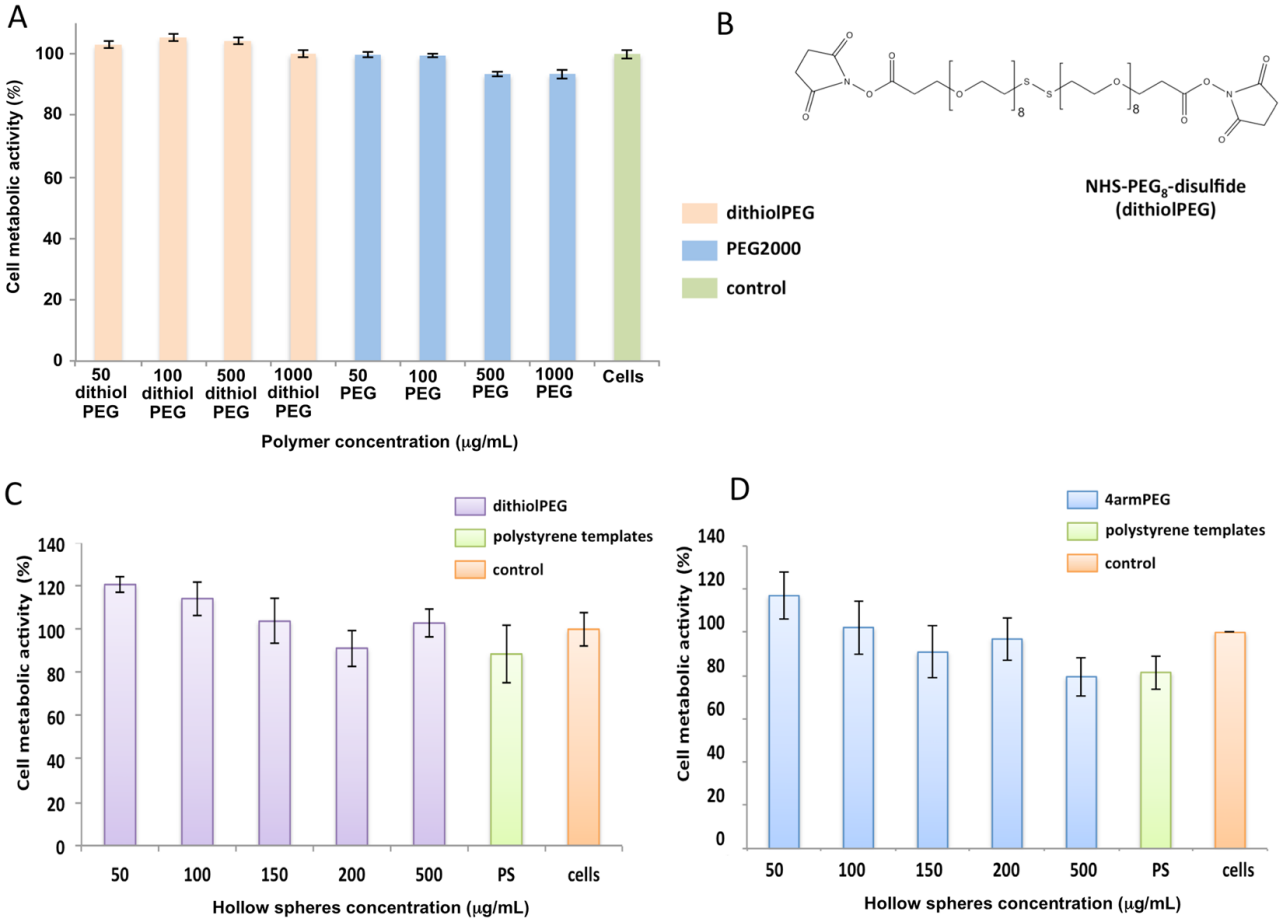
Cell metabolic activity on rat cardiomyoblasts H9C2 treated with control polymers and hollow spheres. (**A**) Linear dithiolPEG, linear PEG2000 after 24 hours of exposure and (**B**) the dithiolPEG chemical structure; alamarBlue® assay was carried out on the dithiolPEG (orange), PEG2000 (blue), and untreated cells (green) (n=3, p<0.05). Cell metabolic activity on the collagen-dithiolPEG hollow spheres (**C**) and collagen-4armPEG hollow spheres (D) (n=3, p<0.05). Polystyrene (green) and untreated cells (orange) were used as controls

The cytotoxicity of the spheres was studied on H9C2 rat cardiomyoblast cells. Cells were exposed to spheres of the same size (1 μm diameter) to determine and compare the cytotoxicity of the constructs. Several doses of each type of hollow spheres (50, 100, 150, 200 and 500 μg/mL) were tested on cells, with the polystyrene template acting as a control. After 24 hours, cells were stained with alamarBlue®, incubated for a further 3 hours and analyzed for metabolic activity. No significant effect on metabolic activity was observed for H9C2 cardiomyoblasts treated with the different types of spheres in comparison to the polystyrene and untreated controls (Figs 3 and 4). Our results in Fig. 3 highlight the toxicity of polypropylene sulfide at higher concentrations, which correlate with reports in the literature^22,23^. The linear polypropylene sulfide polymer can freely diffuse and is internalized within the cells, resulting in significantly reduced cellular metabolic activity. The PPS-collagen hybrid spheres were fabricated by adapting a previously published template method developed in our laboratory^24–26^. The template based method allowed us to engineer hollow spheres with a layer of collagen followed by a layer of the ROS responsive polypropylene sulfide. Precise control of the size of the hollow microspheres is required which can only be achieved using this templating method. The first critical step is the preparation of the template and its functionalization. This is critical as tailoring the final structure (membrane thickness, release) follows^27^. We have previously reported the influence of the size and charge of hollow spheres on cellular internalization and cellular viability^28^. In this study the most stable and optimal concentration ratio of collagen to PPS exhibited no reduction in cellular metabolic activity. Collagen as an extracellular matrix protein provides adhesion sites for cells. We postulate that the remarkably decreased cytotoxicity observed for the collagen-polypropylene sulfide hybrid spheres is due to the high crosslinking efficiency of collagen-PPS, which reduces the exposure of the charged groups of the PPS to the cells (Fig. 3A,B).

In order to validate the metabolic activity and evaluate the suitability of the hollow micro hybrids as a possible drug delivery system, the cellular uptake and cytotoxicity of FITC-labeled hollow spheres *in vitro* on H9C2 cardiomyoblast cells was investigated. For the uptake studies, H9C2 cardiomyoblast cells were incubated with FITC-labelled hollow spheres dispersed at a mass concentration of 50 μg/mL and 100 μg/mL (Fig. 3C and D). An efficient uptake of hollow spheres by the cells after 24 hours of incubation was confirmed by the green signal due to the FITC-labeled hollow spheres in the cytoplasm region. Cytotoxicity control experiments were conducted on mixed collagen-dithiolPEG and mixed collagen-4armPEG hollow spheres and the pristine dithiolPEG synthetic polymer (Fig. 4).

### ROS responsiveness of hollow spheres

**Figure 5:**
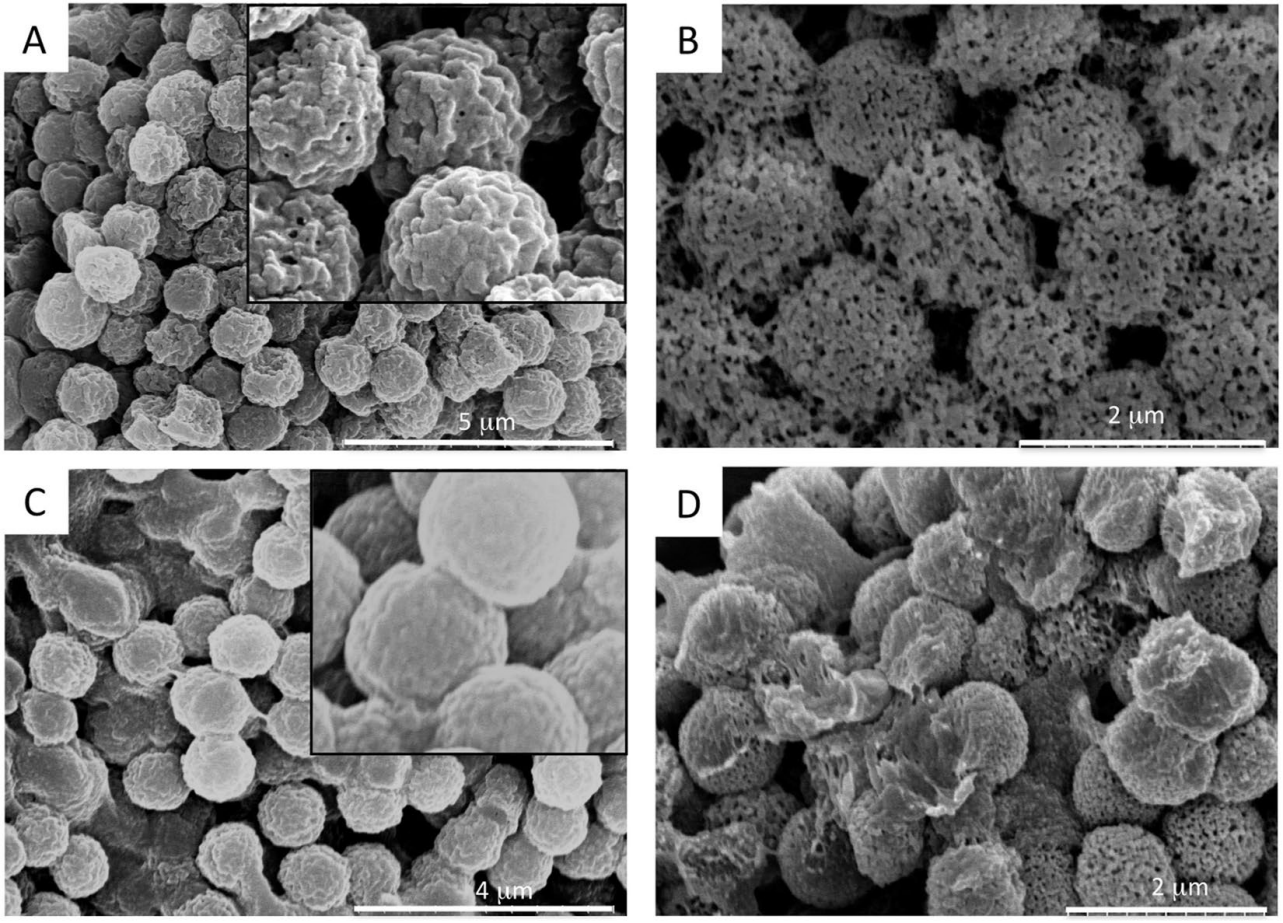
SEM imaging of ROS-responsive hollow spheres. 1 μm collagen-PPS hollow spheres before (**A**), and after (B) 72 hours exposure to H_2_O_2_ (**B**). Control: collagen-dithiolPEG hollow spheres before (**C**), and after (**D**) 72 hours exposure to H_2_O_2._ Inset: zoom views of the hollow spheres surface, prior to H_2_O_2_ exposure.

**Figure 6:**
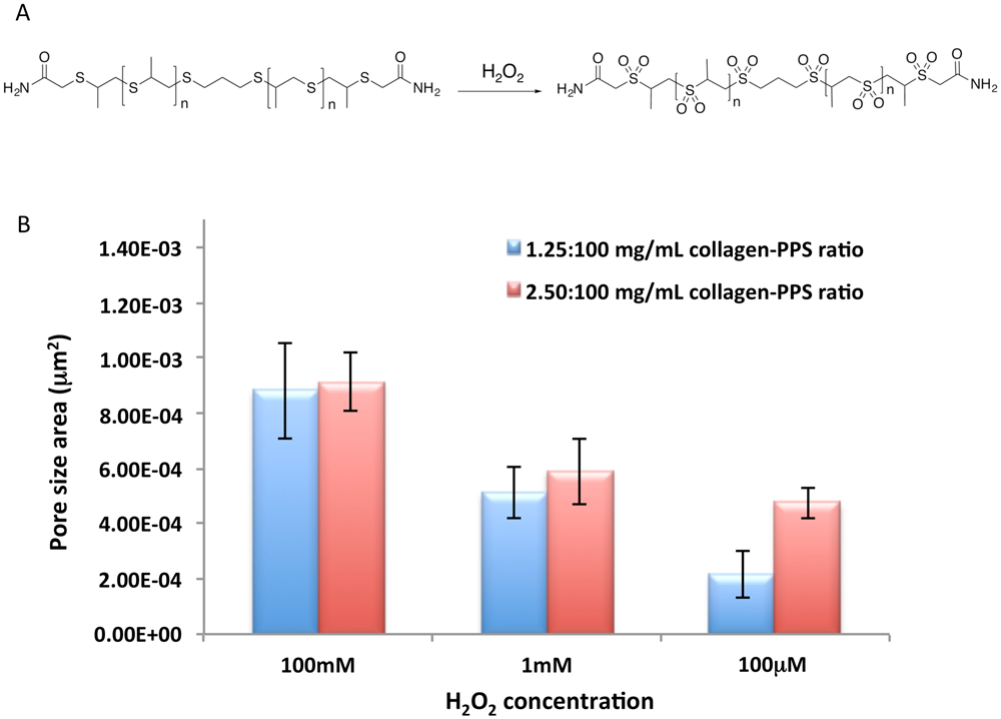
ROS responsiveness quantification. (**A**) Oxidation mechanism of the polypropylene sulfide (PPS) component and (**B**) pore size area distribution of collagen-PPS hollow spheres (evaluated with Image J). All nanoporation analyses were conducted at the 72 hours time point.

The hydrophobic to hydrophilic switch that the polypropylene sulfide undergoes makes it responsive to ROS, while still acting as a protective function for the collagen layer. Structural analysis of the hollow spheres via SEM showed that for the collagen-PPS hollow spheres, a ROS nanoporation phenomenon occurs, with different pore size depending on the H_2_O_2_ concentration exposure over the time points studied (Figs 5A,B and 6B). Conversely, both collagen-4armPEG hollow spheres and collagen-dithiolPEG hollow spheres were completely disrupted by the interaction with H_2_O_2_ (Fig. 5C and D). Consequently, no pore size could be determined in these cases. Thus, the presence of PPS renders hollow spheres sensitive to ROS, but also effectively protects the natural component from massive degradation. This swelling is due to the PPS backbone becoming oxidized, resulting in nanopore formation on the spheroid constructs (Fig. 5B), which become spongy and more permeable to solutes.

Hollow spheres were dried and tested in a powder format for FT-IR analysis. The comparison between polypropylene sulfide and hollow spheres samples by FT-IR analysis confirmed the oxidation of the synthetic component in the final construct (Fig. S13A).

Further examination of the surface of the hollow spheres was performed, evaluating the pore size distribution for different hollow sphere batches, depending on H_2_O_2_ exposure parameters (H_2_O_2_ concentration and exposure time). As confirmed by the FT-IR spectra, the collagen-PPS hollow spheres exhibit ROS responsiveness, in a time and concentration dependent manner. Higher pore surface areas were detected for higher H_2_O_2_ concentrations (Fig. 6B). Samples with a lower amount of collagen (thus a higher amount of PPS) were demonstrated to be more sensitive in terms of nanopore area formation, as a result of H_2_O_2_ exposure. Moreover, the pore area dependence on the concentration of PPS was remarkable at a physiologically relevant concentration (100 μM)^29^.

Thus, the PPS concentration ratio in the hollow spheres micro hybrids fabrication protocols was established and optimized, resulting in a protective action for ROS degradation, which otherwise would have led to structural disruption of the hollow spheres, as observed in the controls (Fig. 5D and S11, S12). Considering that a hollow sphere of 1 μm diameter (0.5 μm radius) has been estimated to present a surface area of 3.14 μm^2^, the pore size of the hollow spheres were demonstrated to almost double as the concentration of H_2_O_2_ was increased (Fig. 6). The 2.5:100 mg/mL ratio, collagen: PPS hollow spheres concentration was observed to be the best performing scaffold in terms of reproducibility and tunable responsiveness to ROS exposure. These results perfectly match with the number of pores, which decreased upon exposure to increasing H_2_O_2_ concentrations. Indeed, a higher ROS stress resulted in wider pore surfaces, with pores starting to merge together, thus decreasing the pore number and increasing the porosity area of the hollow spheres (Fig. S13). This phenomenon is accounted for the dual activity of the synthetic component, versus the natural component: on one side, PPS is able to undergo a solubility switch, responsible for the overall scaffold ROS sensitivity, while on the other hand, PPS exploits a protective action on the collagen component, preserving the natural component which would otherwise experience a massive degradation following ROS exposure (Fig. 5C and D). Nevertheless, the PPS solubility-switch properties allow for the creation of a platform, which exhibits a nanoporation mechanism triggered by ROS exposure.

In conclusion, the synthesised polypropylene sulfide (PPS) exhibited high ROS-responsiveness and this polymeric backbone was subsequently functionalized to achieve an effective conjugation with type I collagen. Moreover, the optimal conditions to fabricate a hybrid natural/synthetic hollow sphere construct were established by using a template method, which guaranteed a monodispersed population. Optimized collagen-PPS hollow spheres exhibited a ROS-triggered nanoporation, with the pore size dependant on the ROS concentration within the physiological range, over a period of 72 hours. In addition, cellular viability as measured by metabolic activity of H9C2 cardiomyoblasts was maintained. The best performing scaffold was demonstrated to be the 2.5:100 mg/mL ratio, collagen-PPS hollow spheres, which was still able to undergo a ROS-triggered solubility switch, while preserving the natural collagen component from a massive degradation following ROS exposure. The composite natural/synthetic hollow microspheres developed are a promising platform that can be harnessed as a pathological stimuli-responsive reservoir for the delivery of therapeutics for the treatment of inflammatory diseases^30^. Future *in vivo* studies are required to assess the responsiveness of the spheres to other ROS including superoxide, hydroxyl radical, hypochlorite ion, hydrogen peroxide and singlet oxygen^31^, to fine tune the platform technology for specific biomedical applications.

## Experimental Section

### Materials

All materials and reagents used in this study were purchased from Sigma Aldrich Ireland Ltd. (Dublin, Ireland), unless otherwise stated. Type 1 atelocollagen was isolated as previously described^32^. Briefly; bovine tendons were blended, washed in buffer and suspended in 0.5 M acetic acid. The resulting solution was then pepsin treated and filtered to remove insoluble collagen telopeptides. The soluble collagen was subsequently purified by repeated salt precipitation and centrifugation, followed by dialysis against 0.01 M acetic acid.

### Polypropylene sulfide synthesis

**Figure 7:**
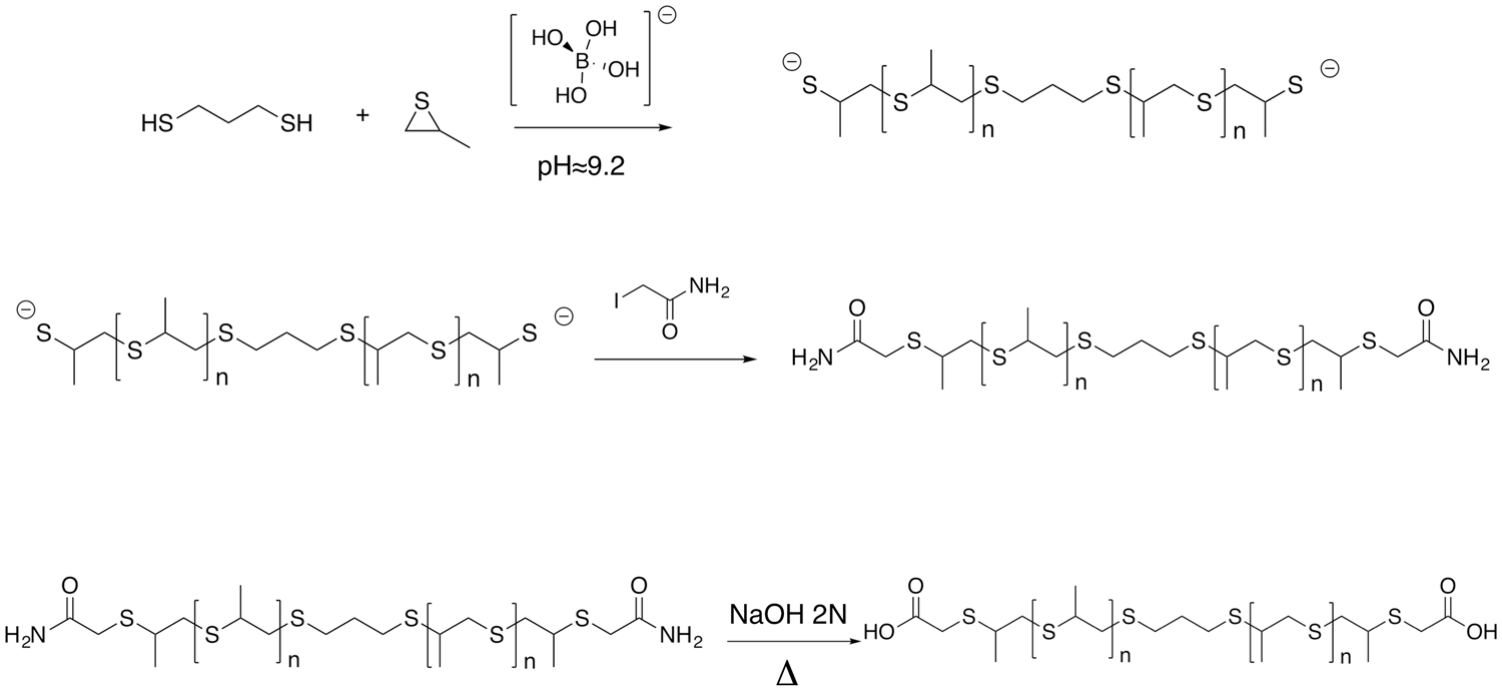
Synthesis of the polypropylene sulfide polymer.

An emulsion polymerization approach was applied^33^. 90 mL of deionized water was degassed, for one hour, stirring at 200 rpm. 1.0 g of Pluronic® F-127 was dissolved in the degassed water, and degassed for an additional 1 hour. The system was kept stirring at 1000 rpm and purged with nitrogen for 20 minutes before polymerization. Thereafter, propylene sulfide (12.76 mmol, 0.946 g, 0.894 mL) and 1,3-propanedithiol (0.13 mmol, 0.014 g, 14.79 μL) were added; the polymerization reaction was initiated by adding 10 mL of borate buffer (pH = 9–9.2). After two hours, the reaction was stopped by adding iodoacetamide as end-capping agent (0.38 mmol, 0.07 g) with stirring for an additional one hour. The emulsion was saturated with NaCl and then extracted twice with 50 mL of dichloromethane. The organic phases were subsequently mixed and dried using sodium sulfate. Sodium sulfate was then removed by filtration. Impurities of Pluronic® F-127 and unreacted monomer were removed by washing twice with methanol. The amide adduct (PPS-CONH_2_) was then hydrolysed to its carboxylic acid under basic conditions, obtaining a polypropylene sulfide-COOH (PPS-COOH), as shown in Fig. 7.

### Characterisation of polypropylene sulphide

^1^H NMR and ^13^C NMR spectra were recorded on a 400 MHz Jeol spectrometer, with field frequency lock on CDCl_3_. FT-IR spectra were recorded in ATR mode on a Varian 610-IR spectrometer. FT-IR and^1^H NMR was conducted before and after ROS exposure as well, to identify the specific signals relating related to oxidation of sulfur bonds. GPC was performed on 1% polymer solutions in water on a Varian 920-LC equipped with refractive index detection, using a universal calibration with linear PEG standards. Solutions were filtered with Millex®-PH13 Fluoropore™ PTFE, 13 mm, 0.5 μm pore size, before injection. Additionally, Pluronic® F-127 on its own was tested to check the successful removal, after methanol extraction. Differential Scanning Calorimeter (DSC-60) TA-60WS Shimadzu was run on the solid form of polypropylene sulfide.

### Fabrication of hollow spheres

Polypropylene sulfide amide was treated in basic conditions to obtain the carboxylic acid adduct. A further purification protocol was applied to remove any possible traces of unreacted materials. The crosslinking approach was based on the condensation among the free-NH_2_ groups of the collagen side chains and the –COOH group of the polypropylene sulfide, enhanced by the use of N-(3-Dimethylaminopropyl)-N′-ethylcarbodiimide hydrochloride (EDC) and N-hydroxysuccinimide (NHS). The reaction was conducted during the hollow spheres fabrication step, at 4 °C, under constant shaking for 24 hours.

### Fabrication of collagen-polypropylene sulfide composite hollow spheres

**Figure 8:**
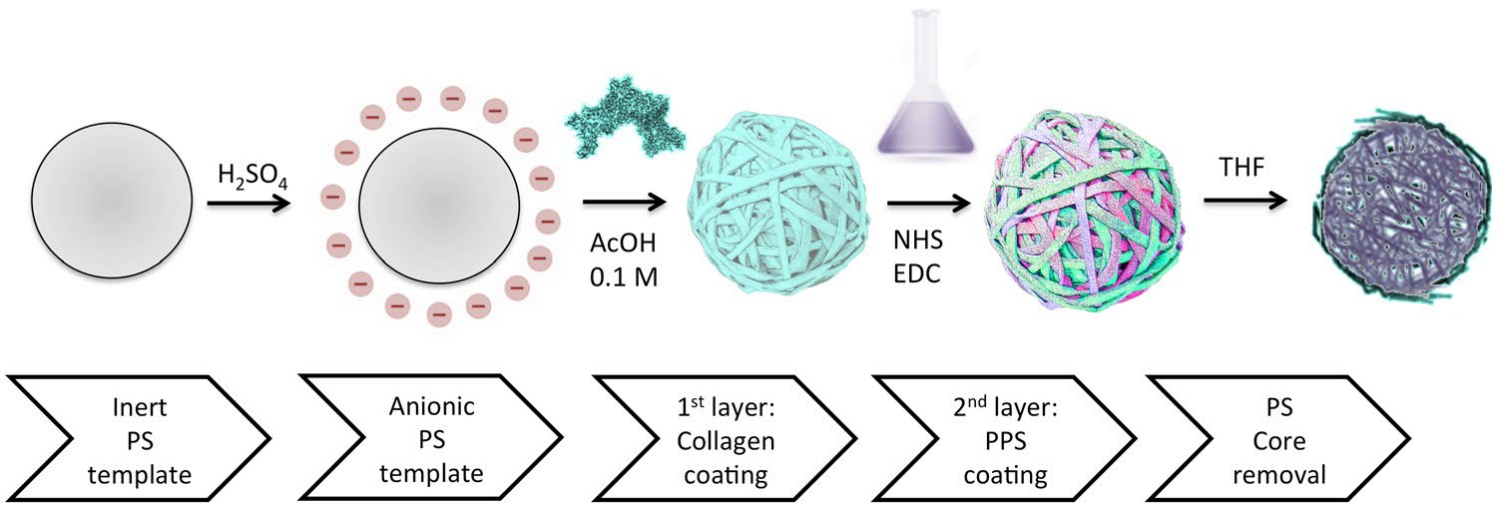
Step-by-step graphical representation of ROS-responsive hollow spheres fabrication

Hollow spheres were fabricated by adapting a template method previously developed in our laboratory^24–26,28,34^, as represented in Fig. 8. Briefly, polystyrene templates (1 μm diameter, Spherotech Inc., US) were treated with sulphuric acid to obtain negatively charged templates. Templates were first coated with a collagen type I layer and subsequently cross-linked with the polythioether carboxylic acid, with a large excess of the cross-linker to ensure effective protection of the collagen from ROS degradation and to provide for a ROS-triggered reaction of the polythioether outer layer. A ratio of 1:7 ratio of collagen to polystyrene was used to ensure that the template was coated with a single collagen layer, with no collagen excess in the medium. Several crosslinking ratios were tested. For clarity, Table 2 selectively reports the concentration ranges, which led to both effective hollow spheres fabrication and related responsiveness. The polystyrene core was then dissolved by tetrahydrofuran (THF) washes to obtain hollow spheres. Collagen-4armPEG composite hollow spheres and collagen-dithiolPEG were used as controls. Collagen type I was dissolved in 0.1 M acetic acid, PPS-COOH was dissolved in methanol, while 4armPEG-COOH and dithiolPEG were dissolved in PBS. To promote the reaction between collagen type I and PPS-COOH or 4armPEG-COOH, N-(3-Dimethylaminopropyl)-N′-ethylcarbodiimide hydrochloride (EDC) and N-Hydroxysuccinimide (NHS) were added during the crosslinking step.

**Table 2:** Experimental concentration ratio ranges for fabrication of composite hollow spheres.

Natural component concentration (Solvent)	Synthetic component concentration (Solvent)	Sample
2.5 mg/mL collagen (0.1 M AcOH)	100 mg/mL PPS-COOH (MeOH)	Construct
1.25 mg/mL collagen (0.1 M AcOH)	100 mg/mL PPS-COOH (MeOH)	
2.5 mg/mL collagen (0.1 M AcOH)	100 mg/mL dithiolPEG-NHS (PBS)	Control
2.5 mg/mL collagen (0.1 M AcOH)	5 mg/mL 4-armPEG (PBS)	

**Figure 9:**
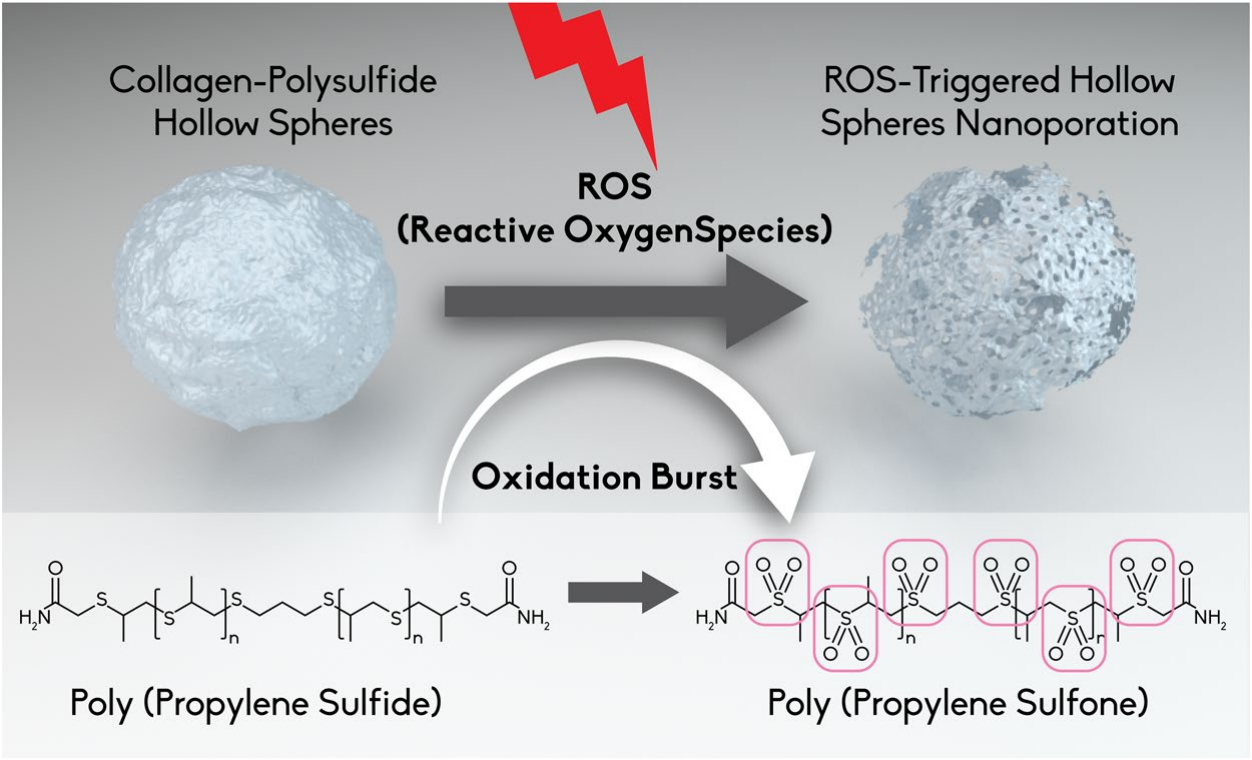
Schematic representation of the nanoporation which the collagen-PPS hollow spheres undergo a in the presence of ROS due to the oxidation-triggered swelling switch of poly(propylene sulfoxide) to poly(propylene sulfone).

A schematic representation of the nanoporation which the collagen-PPS hollow spheres undergo a in the presence of ROS undergoes a ROS-responsive swelling switch is presented in Fig. 9.

### Characterisation of collagen-polypropylene sulfide composite hollow spheres

Following fabrication, the size surface chemical structure and ROS responsiveness of the hollow spheres was evaluated, using scanning electron microscopy (SEM) (Hitachi S-4700 field emission microscope, operating with a beam voltage of 5 kV). A 100 μL aliquot of hollow spheres suspension was pipetted out into a PCR tube, dried overnight, and then deposited on carbon tabs (Agar Scientific), mounted on the SEM specimen stubs. Samples were gold-coated using an Emitech K550 coating system (Derby, England, UK). TEM measurements were performed using a Hitachi H-7500 microscope. Firstly, a 200 mesh TEM carbon-coated copper grids (Agar Scientific) was deposited on a petri dish; the hollow spheres suspension was then carefully deposited (18 μL), and dried overnight at room temperature. The dried samples were then observed under TEM. The mean hollow spheres diameter distribution was determined by dynamic light scattering (photon correlation spectroscopy, PCS) using an N5 Particle Analyzer (Beckman Coulter Inc., USA), equipped with a Peltier temperature control unit. Data was collected at a 90° scattering angle. The time-averaged autocorrelation functions were transformed into intensity-weighted distributions of the apparent hydrodynamic diameter using the available Beckman PCS software. FT-IR spectra were recorded in ATR mode on a Varian 610-IR spectrometer to assess the complete polystyrene core removal and responsiveness to ROS exposure. The surface roughness of the hollow spheres was measured using non-contact mode atomic force microscopy (AFM; Park Systems XE-100).

### Condition of ROS exposure

Hydrogen peroxide (H_2_O_2_) solution at different concentrations was used to mimic the ROS exposure levels at different time points, as shown in Table 1. Aliquots of 100 μL of hollow spheres suspensions were pipetted out and dried overnight. The ROS-treated samples were then resuspended in the H_2_O_2_ solutions and incubated at 37 °C, under continuous shaking to allow H_2_O_2_ permeation.

#### Cytotoxicity assays

The cytotoxicity of the synthesized polypropylene sulfide amide was evaluated on H9C2 rat cardiomyoblasts, by exposing the cells to different polymer concentrations for 24 hours (0.05 mg/mL, 0.1 mg/mL, 0.5 mg/mL, 1 mg/mL). Non-treated cells and cells treated with PEG 2000, as well as with dithiolPEG, were used as controls. The effect of collagen-polypropylene sulfide composite hollow spheres was evaluated after 24 hours exposure at different concentrations (50 μg/mL, 100 μg/mL, 150 μg/mL, 200 μg/mL, 500 μg/mL). Collagen-4armPEG composite hollow spheres, collagen-dithiolPEG, polystyrene templates and untreated cells were used as controls. Cells were seeded in 96-well plates to evaluate metabolic activity by alamarBlue® assay (Invitrogen, Dublin, Ireland).

### Confocal imaging

H9C2 rat cardiomyoblast cells were seeded on 6 well plates with glass slides at a density of 5 × 10^4^ cells/well and cultured overnight in the maintenance medium at 37 °C in a 5% CO_2_ humidified incubator. The following day, cells were treated with 50 μg/mL and 100 μg/mL FITC-labelled hollow spheres for 24 hours. The samples were then washed three times in PBS (0.1 M, pH 7.4), and incubated with DAPI to label the nuclei. Actine fibres were stained with rhodamine phalloidin. Cells were rinsed three times in PBS (0.1 M, pH 7.4). Finally, to analyse the intracellular localization of hollow spheres, the cells were visualized using a laser scanning confocal microscope (Olympus Fluoview 1000 system), with 40x and 60x magnification oil objectives.

### Statistical analysis

All statistical analysis was performed using SPSS Inc., version 22. Values are expressed as the mean ± standard deviation. Data were compared using one-way ANOVA (analysis of variance), followed by Tukey’s multiple comparison tests to determine statistical significance. Data were considered statistically significant at p < 0.05. P-value (p) and number of biological replicates (n) are reported on figure captions, where relevant.

## Electronic supplementary material

Supplementary Information
