# Supplementary material for: Synthetic/ECM-inspired hybrid platform for hollow microcarriers with ROS-triggered nanoporation hallmarks
Source: Sci Rep. 2017 Oct 13;7:13138. doi: 10.1038/s41598-017-13744-y (PMC5640652; doi:10.1038/s41598-017-13744-y)
Supplement: Supplementary file 1 — Supplementary Information [file 41598_2017_13744_MOESM1_ESM.doc]

**Synthetic/ECM-inspired hybrid platform for hollow microcarriers with ROS-triggered nanoporation hallmarks**

Gesmi Milcovich^a^, Paolo Contessotto^a^, Grazia Marsico^a^, Siti Ismail^a^, Abhay Pandit ^a^*

^a^ CÚRAM SFI Centre for Research in Medical Devices , Biomedical Sciences, National University of Ireland, Galway, Ireland

*Corresponding author. E-mail: abhay.pandit@nuigalway.ie

**Supplementary Information**

Content:

Figure S1-S12:

S1. GPC Analysis of the PPS-CONH_2_

S2. DSC of the PPS-CONH_2_

S3. ^1^H NMR of the PPS-CONH_2_

S4. ^13^C NMR of the PPS-CONH_2_

S5. FT-IR of the PPS-CONH_2_

S6. AFM images of hollow spheres, NC-mode error signal

S7. ^1^H NMR of the oxidized PPS-CONH_2_

S8. FT-IR of the oxidized PPS-CONH_2_

S9. SEM of collagen-PPS spheres (2.5 mg/mL to 100 mg/mL ratio) exposed to H_2_O_2_

S10. SEM of collagen-PPS spheres (1.25 mg/mL to 100 mg/mL ratio) exposed to H_2_O_2_

S11. SEM of collagen-dithiolPEG spheres exposed to H_2_O_2_

S12. SEM of collagen-armPEG spheres exposed to H_2_O_2_

S13. (A) FT-IR of oxidized PPS, mixed collagen-PPS hollow spheres and oxidized control hollow spheres (mixed collagen-dithiolPEG and collagen-armPEG) and (B) Pore number distribution on collagen-PPS hollow spheres exposed to H_2_O_2_.

**Figures S1-S12.**

**GPC analysis of polypropylene sulfide**


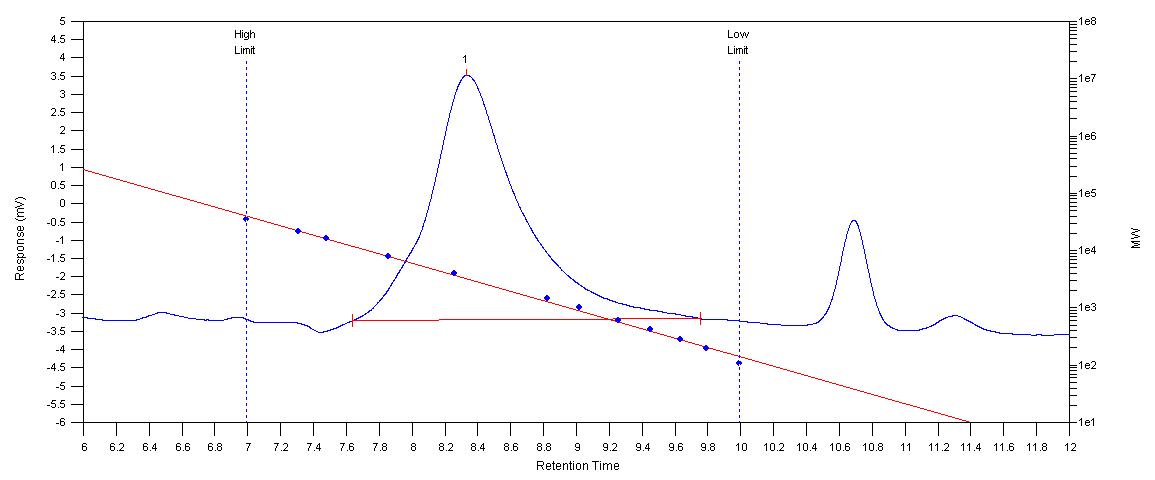

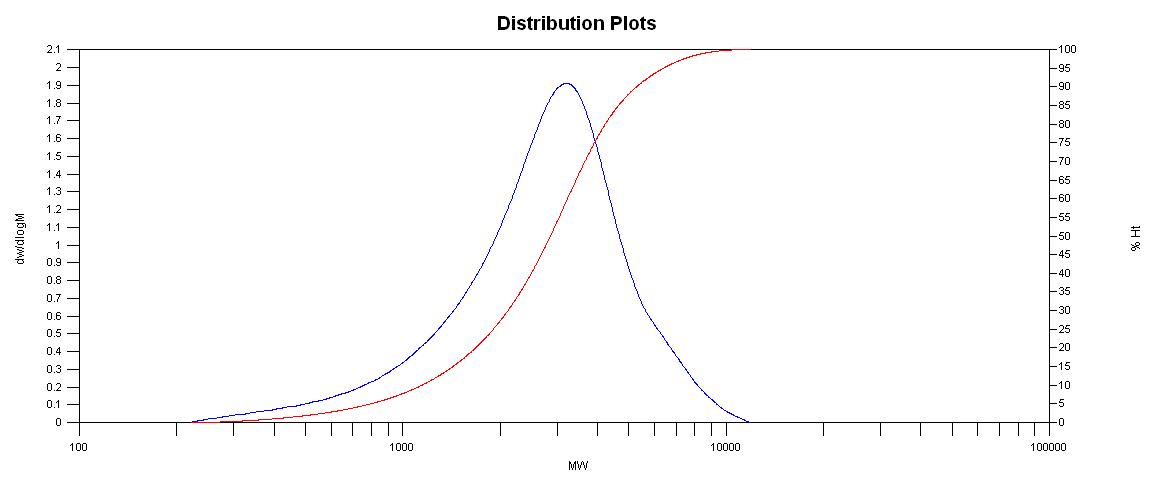


**Figure S1:** GPC trace of polythioether-CONH_2_ calibrated against PEG standards.

**Thermal behavior of the polypropylene sulfide**


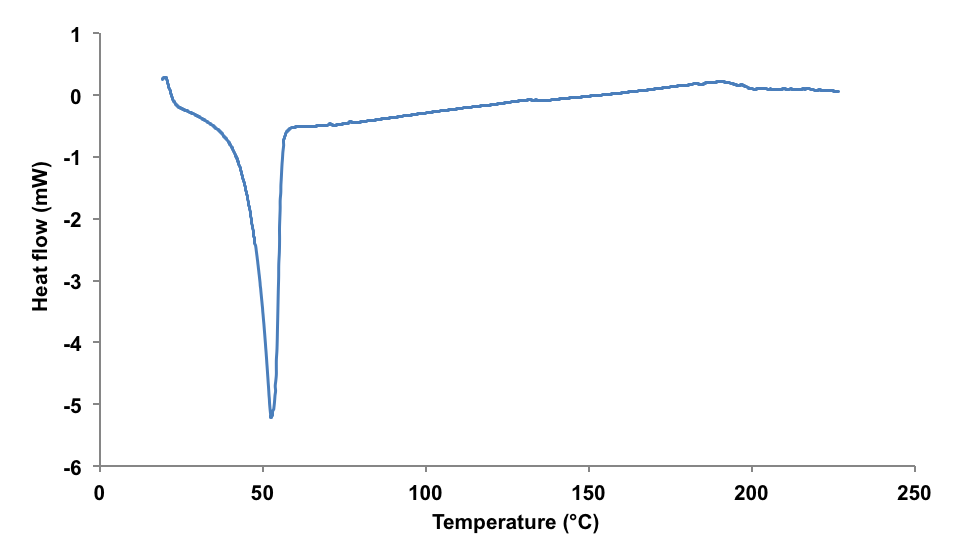


**Figure S2:** Differential Scanning Calorimetry (Melting point: 50.81°C).

**Figure S3:** ^1^H NMR spectrum (CDCl_3_): δ ) 1.33-1.54 (CH_3_ in PPS chain), 1.88 (q, 2H, in -S-CH_2_-**CH_2_**-CH_2_-S-), 2.65-2.66 (CH in PPS chain), 2.85-2.90 (m, CH_2_ in PPS chain), 3.15-3.25 (d, -**CH_2_**-CONH_2_), 6.31 ppm (-NH_2_). Reference on CDCl_3_ (7.22ppm).

**Figure S4:** ^13^C NMR of the PPS-CONH_2_ (400 MHz, Chloroform-d)


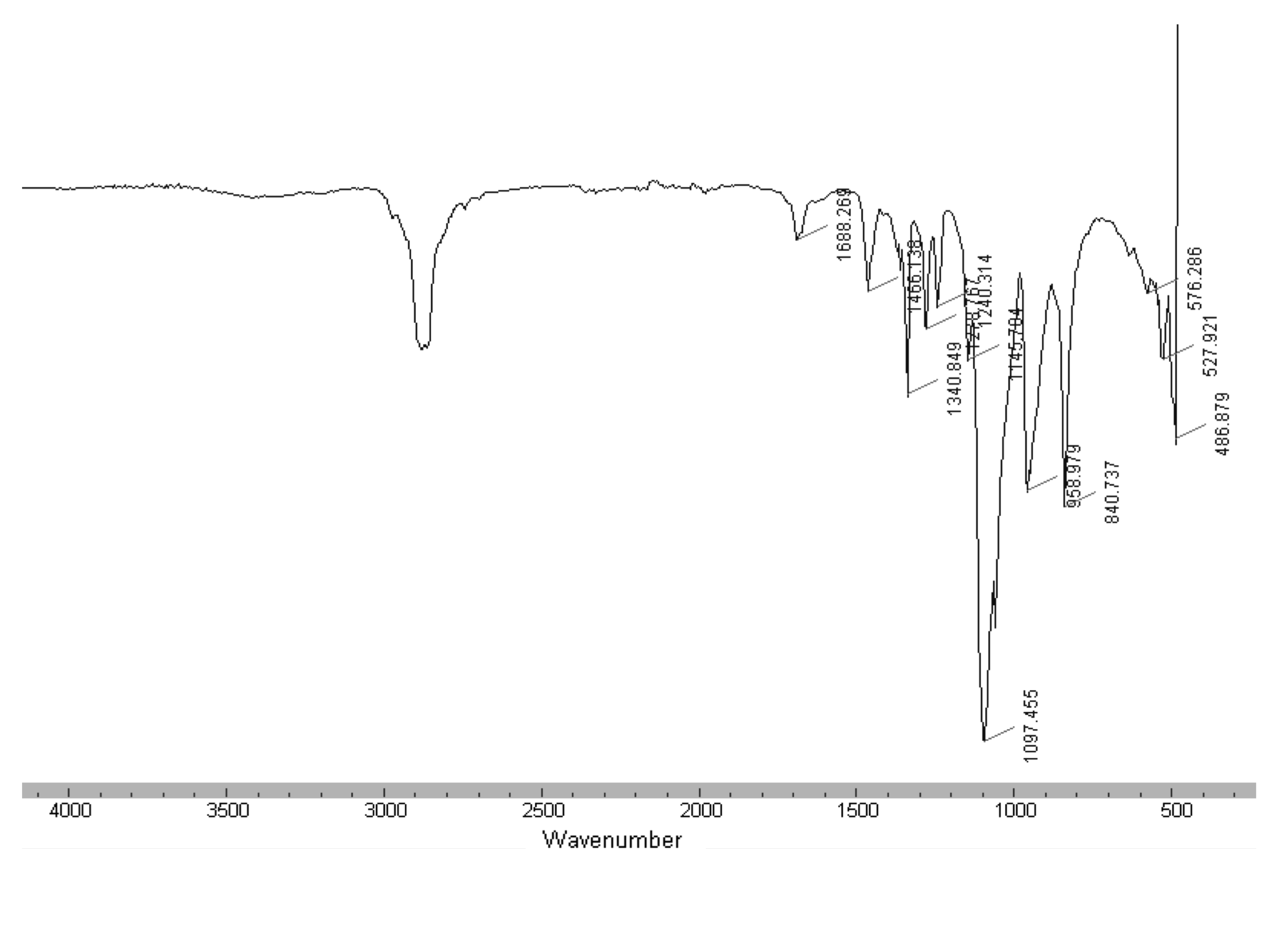


**Figure S5:** FT-IR (powder): 3478.54 cm^-1^(ν_as_ NH), 2879.92 cm^-1^ (ν_s_ CH), 1688.26 cm^-1^ (ν C=O, amide I band), 1466.13 cm^-1^ (ν C-N), 1097.455 cm^-1^ (ν_as_ C-O-C in Pluronic residues), 840.737 cm^-1^ (ν C-S).


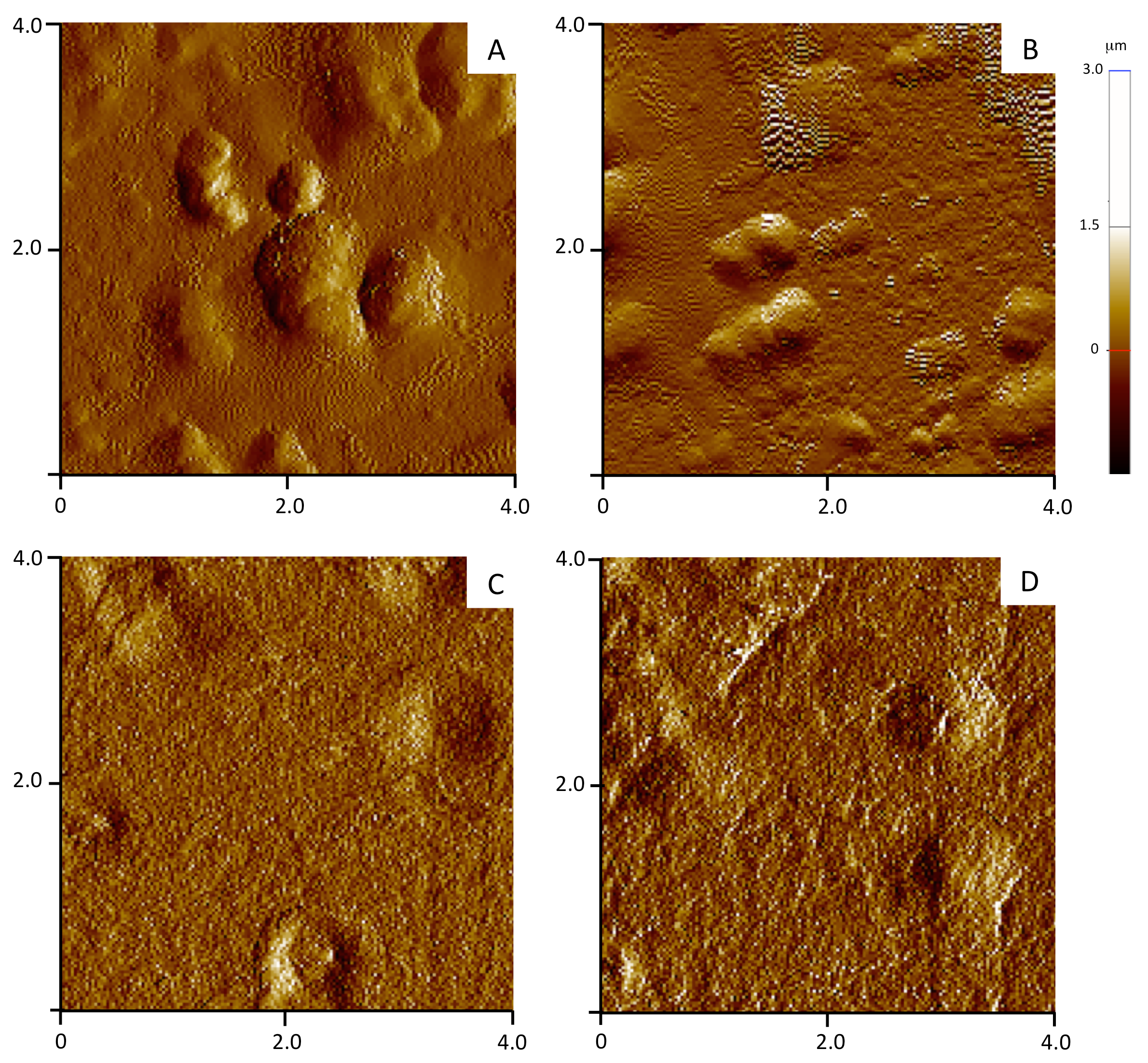


**Figure S6:** AFM NC-mode error signal images of (A) mixed collagen-PPS hollow spheres, (2.5 mg/mL to 100 mg/mL ratio), (B) mixed collagen-PPS hollow spheres, (1.25 mg/mL to 100 mg/mL ratio), (C) mixed collagen-dithiolPEG hollow spheres (control) and (D) mixed collagen-4armPEG hollow spheres (control).

**Figure S7:** ^1^HNMR of PPS-CONH_2_ after H_2_O_2_ exposure, drying and resuspend in CDCl_3_. Peaks labeled in red correspond to the chemical shifts related to the oxidation of thioether bonds to sulfone.

**Figure S8:** FT-IR (powder) after H_2_O_2_ exposure of the PPS-CONH_2_ synthesized compound (100 mM H_2_O_2_ for 24 h). 3236.45 cm^-1^(ν_as_ NH), 2944.59 cm^-1^ (ν_s_ CH), 1642.45 cm^-1^ (ν C=O, amide I band), 1461.00 cm^-1^ (ν C-N), 1133.95 cm^-1^ (ν_s_ O=S=O), 632.00 cm^-1^ (ν C-S).

**Figure S9:** Hollow spheres responsiveness to ROS. Clockwise: 1 μm collagen-PPS hollow spheres (2.5 mg/mL to 100 mg/mL ratio) before (A), and after 72 h H_2_O_2_ exposure at 100 mM(B), 1 μM (C), and 100 μM (D).

**Figure S10:** Hollow spheres responsiveness to ROS. Clockwise: 1 μm collagen-PPS hollow spheres (1.25 mg/mL to 100 mg/mL ratio) before (A), and after 72 h H_2_O_2_ exposure at 100 mM(B), 1 μM (C), and 100 μM (D).

**Figure S11**. Hollow spheres responsiveness to ROS. Clockwise: 1 μm collagen-dithiolPEG hollow spheres (2.5 mg/mL to 100 mg/mL ratio) before (A), and after 72 h H_2_O_2_ exposure at 100 mM (B), 1 μM (C), and 100 μM (D).

**Figure S12:** Hollow spheres responsiveness to ROS. Clockwise: 1 μm collagen-armPEG hollow spheres before (A), and after 72 h H_2_O_2_ exposure at 100 mM (B), 1 μM (C), and 100 μM (D).

**Figure S13:** (A) FT-IR spectra comparison among oxidized PPS (red), mixed collagen-PPS hollow spheres (2.5:100 mg/mL ratio) (green) and mixed collagen-PPS hollow spheres (1.25:100 mg/mL ratio) (violet), oxidized mixed collagen- dithiolPEG (light blue) and collagen-4armPEG (black) hollow spheres. All samples were treated with H2O2 for 72h. (B) Pore number distribution on collagen-PPS hollow spheres (evaluated with Image J). All nanoporation analyses were conducted at the 72h time point.
